# Transcript Diversification in the Nervous System: A to I RNA Editing in CNS Function and Disease Development

**DOI:** 10.3389/fnins.2012.00099

**Published:** 2012-07-09

**Authors:** Aamira Tariq, Michael F. Jantsch

**Affiliations:** ^1^Max F. Perutz Laboratories, Department of Chromosome Biology, University of ViennaVienna, Austria

**Keywords:** calcium channel, glutamate receptor, inosine, potassium channel, RNA modification, RNA editing, serotonin receptor

## Abstract

RNA editing by adenosine deaminases that act on RNA converts adenosines to inosines in coding and non-coding regions of mRNAs. Inosines are interpreted as guanosines and hence, this type of editing can change codons, alter splice patterns, or influence the fate of an RNA. A to I editing is most abundant in the central nervous system (CNS). Here, targets for this type of nucleotide modification frequently encode receptors and channels. In many cases, the editing-induced amino acid exchanges alter the properties of the receptors and channels. Consistently, changes in editing patterns are frequently found associated with diseases of the CNS. In this review we describe the mechanisms of RNA editing and focus on target mRNAs of editing that are functionally relevant to normal and aberrant CNS activity.

## RNA Editing

RNA editing is a site specific, post transcriptional modification of RNA. Two types of RNA editing can be distinguished. On the one hand, insertion-deletion type editing inserts or removes single or multiple nucleotides from an RNA molecule (Simpson et al., 2000). This type of editing is mostly found in organelles of various phyla. Deamination type editing, on the other hand, changes the identity of a base by deaminating cytidine to uracil or adenosine to inosine, respectively (Carter, 1995). Deamination type editing has been found in all kingdoms of life. Deamination of cytidines was first discovered in the mRNA encoding apolipoprotein B which is deaminated by Apobec1 a member of the apobec/AID cytidine deaminase family that mostly target cytidines in DNA. Recently, abundant cytidine deamination by Apobec1 was identified in the 3′ UTRs of many mouse mRNAs (Rosenberg et al., 2011). The function of these editing events remains to be determined, however.

Adenosine deamination by adenosine deaminases that act on RNA (ADAR) seemingly only affects metazoan nuclear encoded RNAs. Deamination of adenosines leads to the formation of inosines which are recognized as guanosines by most cellular machineries (Bass and Weintraub, 1988). Editing in coding regions of mRNAs can therefore lead to a codon exchange and the subsequent translation of a protein that differs from the genomically encoded version. Moreover, editing can also introduce or remove splice sites and thereby lead to the formation of novel mRNAs (Rueter et al., 1999). Finally, inosines in RNA can change the base-pairing propensity of an RNA and therefore alter their folding and change their signature for RNA-binding proteins (Nishikura, 1992). Thus, also editing in non-translated regions of an mRNA may have profound impact on the fate of the affected RNA. Besides mRNAs, also primary and precursor miRNAs can be targets for RNA editing by ADARs. Editing of pri- and pre-miRNAs can alter their processing but also their base-pairing potential with target mRNAs. Therefore, editing of miRNAs can indirectly change the abundance and translatability of their target mRNAs.

Editing of mRNAs was originally believed to be a rare event. In recent years, however, editing was found to be widespread in mRNAs of higher eukaryotes (Athanasiadis et al., 2004; Levanon et al., 2004). In all organisms editing by ADARs is most abundant in the nervous system. The profound alterations of the transcriptome and proteome introduced by RNA editing may thus help to solve a long lasting biological paradigm, namely, how biological complexity can be achieved with an almost constant number of genes: editing-induced alterations of splice patterns and coding potential of mRNAs may, together with alternative splicing, contribute to the formation of a complex proteome from a limited number of genes. Consistently, alterations in the editing patterns or loss of editing is accompanied by pathologic conditions and disease (Morabito and Emeson, 2009).

## The ADAR Protein Family

ADARs were first discovered in *Xenopus laevis* as an unwinding activity that destabilizes RNA duplexes upon A to I editing (Bass and Weintraub, 1987; Rebagliati and Melton, 1987). ADARs have been well characterized in many organisms including insects, worms, and vertebrates (Bass, 2002). The first ADAR gene identified was vertebrate ADAR1 harboring three double-stranded RNA-binding domains (dsRBDs) and a conserved deaminase domain with zinc binding motifs. Subsequent screens led to the identification of ADAR2 (Melcher et al., 1996; O’Connell et al., 1997). Recent analyses have shown the presence of ADAR1 and ADAR2 in many species including sea urchin and sea anemones (Jin et al., 2009). The vertebrate genome encodes two additional, ADAR proteins. ADAR3, which presumably arose from ADAR2 by gene duplication, contains all functional domains. However, no function has been ascribed to this isoform (Chen et al., 2000). The fourth ADAR-like gene, termed TENR, is expressed in the male germ line and has one dsRBD. TENR lacks conserved zinc chelating residues in the deaminase domain thus explaining its inactivity (Hough and Bass, 1997). ADARs are related to the tRNA editing family of ADATs which are found in all kingdoms of life (Jin et al., 2009).

## Phenotypes of ADAR Deficiency

Different phenotypes are associated with the lack of individual ADAR isoforms. ADAR2 null mice have episodes of epileptic seizures and show subsequent postnatal death. A key substrate of ADAR 2 is the mRNA encoding GluA2. Underedited GluA2 allows increased influx of Ca^2+^ leading to death of neurons (Brusa et al., 1995). Consistently, *ADAR2^−/−^*mice can be rescued by replacing the genomic, unedited GluA2 copy with a “preedited” gene copy (Higuchi et al., 2000). Still, even the rescued *ADAR2^−/−^*mice display a range of subtle phenotypes ranging from a decreased acoustic startle response to decreased blood glucose level. The molecular mechanisms underlying these changes are still to be determined (Horsch et al., 2011). ADAR2 overexpressing mice, in contrast, display hyperphagia and obesity (Singh et al., 2007). This phenotype can be reproduced by a catalytically inactive version of ADAR2 that retains its RNA-binding ability. This suggests that RNA binding of ADARs can lead to editing-independent phenotypes (Singh et al., 2007).

Mice lacking ADAR1 die during embryonic development, show defective hematopoiesis, widespread apoptosis, and liver disintegration (Hartner et al., 2004). Molecularly, ADAR1 deficient mice show an increase in interferon signaling with the precise molecular mechanisms leading to death remaining unknown (Hartner et al., 2009).

In *Drosophila melanogaster* inactivation of the single ADAR gene causes tremors, lack of coordination, mating defects, and neurodegeneration presumably resulting from underediting of important dADAR target genes such as Na^+^ (*para*), Ca^2+^(*cac*), and glutamate-gated Cl^−^ channels (*DrosGluCl-*α; Palladino et al., 2000). *Caenorhabditis elegans* strains with homozygous deletions in either of the two ADARs *adr-1* or *adr-2* show chemotactic defects, also indicating a role in the editing of neuronally expressed substrates (Tonkin et al., 2002).

## Substrates of ADAR

RNA editing by adenosine deaminases can affect coding and non-coding RNA sequences. Substrate RNAs are recognized by the dsRBDs located in ADARs. These domains bind to A-form helices formed by double-stranded RNAs. Thus, editing sites are defined by base-paired regions of 20 or more nucleotides in length. A-form helices display a wide minor groove and a narrow major groove. Sequence specific information of the bases cannot be easily contacted making a sequence specific positioning of ADARs difficult (Ryter and Schultz, 1998). Still, various mechanisms can contribute to substrate and editing specificity. Multiple dsRBDs found in ADARs can coordinately bind to substrates (Stefl et al., 2005). Most double-stranded structures formed by endogenous RNAs are disrupted by bulges. These bulges set natural boundaries for the binding of dsRBDs (Lehmann and Bass, 1999). If two or more dsRBDs need to bind to a double-stranded region of limited length they can help to position each other. Structural analysis of ADAR2 bound to a stem loop substrate shows nicely that some dsRBDs can also bind to terminal loops thus helping to increase substrate specific binding (Stefl et al., 2006). Recently, specific minor groove interactions between dsRBDs and nucleotides have been identified. These interactions can increase sequence specificity dramatically, therefore aiding in selecting specific adenosines within a stretch of double-stranded RNA (Stefl et al., 2010). Finally, also the deaminase domains of ADAR1 and ADAR2 display substrate specificities that preferentially select certain adenosines depending on their local sequence context (Polson and Bass, 1994; Eggington et al., 2011). As the adenosine to be edited typically lies within a double-stranded structure the target adenosine needs to be accessed through a base flipping mechanism (Stephens et al., 2000; Yi-Brunozzi et al., 2001).

The altered base-pairing potential of inosines can lead to an alteration of the RNA secondary structure. Thus, editing in the non-translated regions of mRNAs may alter their localization, stability, and translatability. However, the biological consequences of editing in these targets is still under debate. The consequences of adenosine deamination in coding regions of mRNAs and pri-miRNAs on the other hand are more easy to understand. As inosines are read as guanosines during translation, inosines can alter the coding potential or targeting specificity of mRNAs and miRNAs, respectively (Vesely et al., 2012).

The proteins encoded by edited pre-mRNAs vary widely in their function. However, frequently editing-induced amino acid exchanges affect receptors and ion channels expressed in the brain. Another class of proteins affected by RNA editing play a role in cytoskeletal remodeling which also plays an important role in neuronal outgrowth and plasticity. In the following, representative examples of both classes of proteins and the functional implications of their editing will be described.

## Glutamate-Gated Ion Channels

Five subunits of the glutamate receptor (GluA2, GluA3, GluA4, GluK1, and GluK2) are found to undergo ADAR-mediated RNA editing (Bass, 2002). A total of four editing sites that result in amino acid changes have been identified, namely glutamine to arginine (Q/R), arginine to glycine (R/G), isoleucine to valine (I/V), and tyrosine to cysteine (Y/C; see Table 1).

**Table 1:** **Selected editing events in the CNS**.

Target	Editing site	Function	Diseases
GluA2	Q607 R	Calcium impermeable ER exit efficiency reduction	ALS, epilepsy, glioblastomamultiforme, pediatric astrocytoma
GluA2	R764G	Enhanced rate of desensitization	Spinal cord injury (SCI), epilepsy
		Modulation of alternative splicing	Schizophrenia on drug administration
Serotonin	I156V	Modulation of surface expression of the receptor	Schizophrenia
receptor	I156M	Reduced G protein coupling	Bipolar disorder
5HT2C	N158S	Decreased Erk signaling	Depression
	N158G		Anxiety
	N158D		Prader–Willi syndrome
	I160V		
Kv1.1	I400V	Faster recovery from inactivation	Epilepsy
		Reduced potency of channel blockers	
GABA_A_	I342M	Reduced stability of α3 subunit	Migraine
FLNa	Q2341R	Binds to: Kv4.2 K^+^ Channel	
		Presenilins metabotropicmGlu5a/b, mGlu7b, mGlu8a, weak: mGlu7a mGlu4a	
CyFIP2	K320E		
Nova-1	S383G	Increase in protein stability	
Ca(v)1.3	I1606M	Decrease in calmodulin mediated calcium dependent	
	Q1607R	inhibition (CDI) and faster recovery from inactivation	
	Y1609C	

*Proposed physiological and pathological consequences*.

AMPA GluA2 subunit mRNA was the first target discovered. It is edited mainly at two coding sites leading to a glutamine to arginine and arginine to glycine conversion (Sommer et al., 1991; Lomeli et al., 1994; see Figure 2).

**Figure 1:**
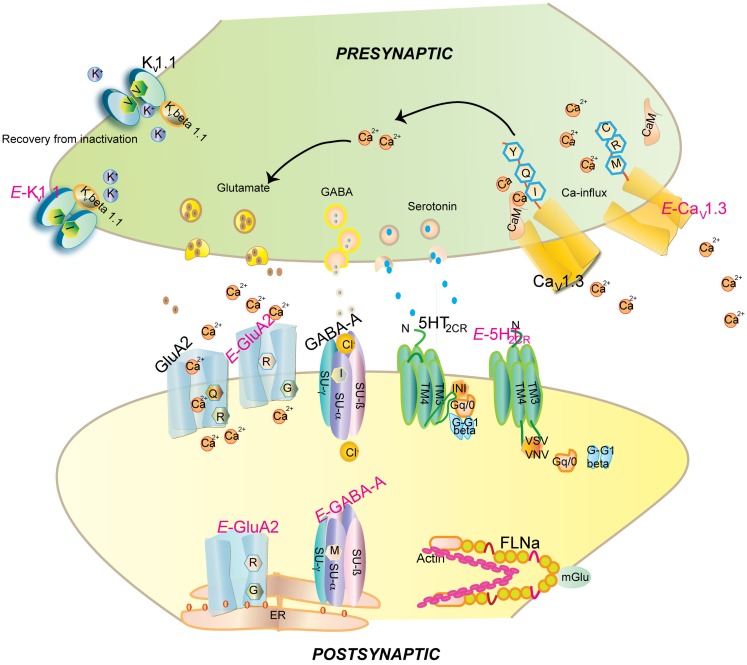
**Impact of editing on selected neuronal receptors and proteins**. Shown are several receptors and channels in their unedited (black) and edited (E, in pink) versions. Editing at the Q/R site of ionotropic glutamate receptor GluA2 subunit decreases Ca^2+^permeability and endoplasmic reticulum exit efficiency. Membrane trafficking of the GABA_A_ receptor is reduced by editing of I to M in the alpha3 subunit. Editing of the serotonin 5-HT_2c_ receptor converts the amino acids I-N-I to V-S-V or V-N-V. This reduces G-protein coupling in the receptor. The editing-induced I to V exchange in K_v_1.1 (I/V) alters the interaction with Kv β1.1 (see Figure 3 for detail). Editing of the IQ motiv in Ca_v_1.3 to MR abolishes calmodulin binding. Filamin alpha (FLNa) is edited in a region that is known to interact with metabotropic glutamate receptor mGlu7b and some of its relatives.

Two additional editing sites are found in intron 11 of GluA2 mRNA, called hotspot 1 (or +60 site) and hotspot 2 (or+262/263/264 site), respectively (Higuchi et al., 1993). Editing at the Q/R site reduces Ca^2+^ permeability (see Figure 1). The edited GluA2^R^ isoforms also show reduced endoplasmic reticulum (ER) exit efficiency, whereas unedited GluA2^Q^ isoforms readily tetramerize and are transported to the synaptic membrane (Greger et al., 2002, 2003). GluA2 in the unedited Q form leads to epileptic seizures and subsequent postnatal death. This toxic effect has been attributed to increased calcium influx (Higuchi et al., 2000). Additionally increased receptor density due to faster ER exit may also contribute to this effect (Greger et al., 2002; see Figure 1).

The R/G conversion reduces the assembly of homomeric receptors and slows down receptor maturation in the ER (Greger et al., 2006). Additionally, R/G site editing results in enhanced recovery from desensitization (Lomeli et al., 1994). Editing events in the GluA2 pre-mRNA also affect splicing of nearby introns. Editing at the R/G site of GluA2 takes place two nucleotides upstream of the 5′ splice site in intron13. The Q/R site is located in exon 11, 25 nucleotides upstream of the 5′ splice site of intron 11 (Higuchi et al., 1993). Editing at the Q/R site and the intronic hotspot enhances splicing of the nearby intron, while editing at the R/G site represses splicing at the downstream intron (Schoft et al., 2007). Editing at the R/G site may affect base-pairing of the pre-mRNA with the U1snRNA (Schoft et al., 2007). R/G site editing also influences the alternative splicing of the two downstream exons as editing promotes inclusion of exon15 (flip) over exon14 (flop). GluA2 protein with an edited G and the flip variant undergoes rapid maturation in the ER relative to the flop form. The flip variant also stimulates dendritic growth (Hamad et al., 2011). The flop isoform, in turn, promotes assembly of heteromeric AMPA receptors (Penn and Greger, 2009).

Also kainate receptor subunits GluK1 and -2 are edited at the Q/R position. GluK2 undergoes additional editing at the I/V and Y/C sites, located at positions 621, 567, and 571 respectively, which may lead to higher calcium permeability (Kohler et al., 1993).

## GABA_A_ Receptor

**Figure 2:**
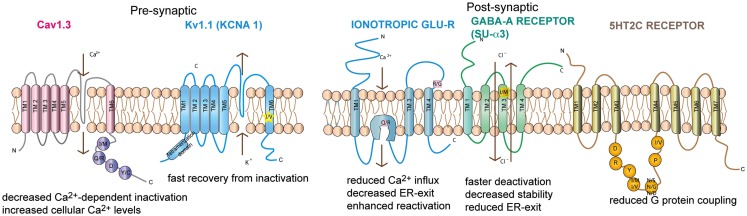
**Topography of the neuronal channels containing editing sites Ionotropic glutamate receptor, GABA_A_ receptor subunit α3, Kv1.1, Cav1.3, and 5-HT_2c_ receptors are shown**. The relative position of the editing sites within these receptors are highlighted. Consequences of editing are indicated.

GABA_A_ receptors are ligand gated chloride channels consisting of five subunits: 2 α subunits, 2 β subunits, and either a γ or a δ subunit (Hevers and Luddens, 1998). The existence of 6 α, 3 β, 3 γ, and 4 δ subunits allows for the assembly of a wide variety of stoichometries. Position 342 of the α_3_ subunit is highly edited, resulting in an isoleucine (AUA) to methionine (AUI) codon change (Ohlson et al., 2007; see Figure 2). The editing site is defined by a specific RNA structure marked by bulges at a defined distance from the editing site as well as a specific terminal loop structure (Tian et al., 2011). With age, the two α subunits show opposing expression patterns. While α_1_ expression increases with age the α_3_ subunit is predominant at embryonic level (Hutcheon et al., 2004). Moreover, editing is developmentally regulated. The pre-mRNA is mostly found unedited around day e15 but is edited from 80% to 100% at postnatal day 7 (p7; Ohlson et al., 2007; Rula et al., 2008)

The I/M change in GABA_A_ receptor causes a delay in currents and faster deactivation upon stimulation by GABA (Rula et al., 2008). Expression of unedited GABA_A_ receptor in the developing brain is crucial for synapse formation (Ben-Ari et al., 2007). Recently, editing has been proposed to affect the stability of the α_3_ subunit as the edited version displays low cell surface expression. The M version of the receptor maintains the hydrophobic environment but can influence the interaction between α and γ subunits or ligand interaction (Daniel et al., 2011; see Table 1; Figure 1).

## Voltage-Gated Potassium Channels

Neuronal Kv1.1 channels are built of a tetramer of pore forming α subunits along with four regulatory beta subunits and accessory subunits. The channels regulate action potential and modulate neuronal excitability by opening and closing of a potassium selective pore. The human Kv1.1 (*KCNA1)* gene is intronless and undergoes A to I RNA editing leading to an isoleucine to valine exchange (see Figure 1). The amino acid exchange is located within the sixth transmembrane segment (S6) which lies at the ion conducting pore (Bhalla et al., 2004; see Figure 2). Kv1.1 channels are edited up to 65–80% in medulla, thalamus and spinal cord (Decher et al., 2010). The I–V change is evolutionarily conserved and also occurs in Kv2 (DmShab Shaker) channels in *Drosophila melanogaster* together with four other editing events (Bhalla et al., 2004; Ryan et al., 2008).

**Figure 3:**
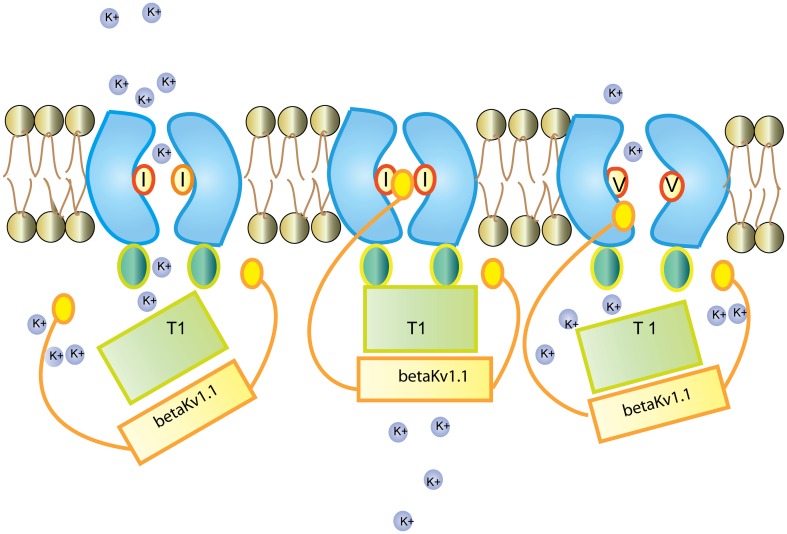
**Regulation of the Kv1.1 channel by K_v_ β1.1 K_v_ β1.1 has an inactivation gate which interacts with the unedited (I) form of the K_v_1.1 receptor**. Editing of K_v_1.1 changes the isoleucine to valine (V). This reduces the affinity for K_v_ β1.1 and enhances recovery from inactivation. Figure adapted from (Bezanilla, 2004).

Kv1.1 associates with the redox sensor Kv β1 in the ER (Pan et al., 2008). Kv β harbors an N-terminal inactivation domain that controls inactivation and lag time of Kv1.1. The edited Kv1.1 shows a 20 fold higher recovery from Kv β1 mediated inactivation than the unedited version of the channel (Bezanilla, 2004; Bhalla et al., 2004; see Figure 3).

The Kv channel blocker 4-aminopyridine (4-AP) has been shown to induce epileptic seizures. RNA editing makes the channel insensitive to 4-AP by disrupting the interaction between the pore lining and the channel blocker (see Table 1; Streit et al., 2011). A similar insensitivity was observed against arachidonic acid (Decher et al., 2010).

Squid Kv 1.1A has also been shown to be extensively edited (Rosenthal and Bezanilla, 2002). Here editing not only affects channel gating but also influences the tetramerization of the channel.

## Voltage-gated Calcium channels

Voltage-gated calcium channels (VGCC) are classified into two types: Low voltage activated (LVA) and High voltage activated (HVA) channels (Lacinova, 2005). LVA l-type calcium channels are involved in a broad range of neuronal processes such as neuronal pacemaking, secretion of neurotransmitters, synaptic transmission, mRNA stability, and modulation of other ion channels (Singh et al., 2008). The opening of these channels permits calcium influx. The channels are inactivated by voltage dependent inhibition (VDI) and intracellular calcium dependent inhibition (CDI). The pore forming α1 subunit contains four domains (I–IV), each domain consisting of six transmembrane segments (S1–S6; Catterall et al., 2005; see Figure 2). S5 and S6 of all four domains form the central pore with S6 lining the inner surface of the pore and occluding the pore in the inactive state. S1 to S4 from each domain form the voltage sensing domain and on activation the S4 segment moves outward triggering S6 movement leading to gate opening (Swartz, 2004).

**Figure 4:**
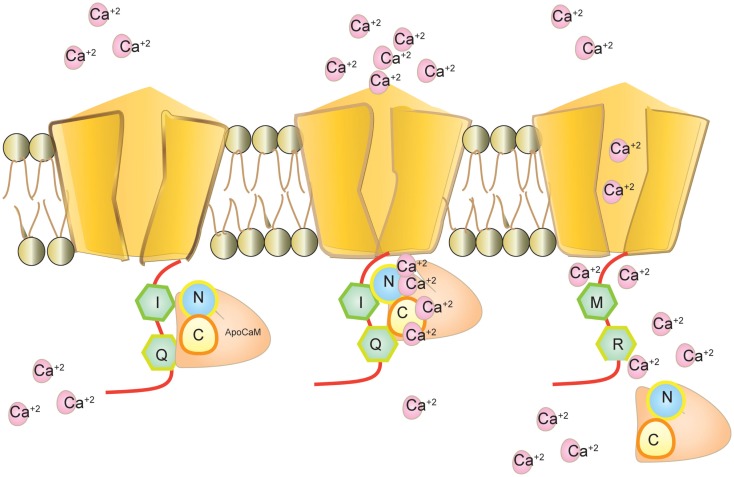
**Ca_v_1.3 and calmodulin interaction**. Calmodulin binds to Ca_v_1.3 without calcium as apoCaM at the IQ motif. Calmodulin binds to calcium through its N-terminal and C-terminal loop and mediates calcium dependent inhibition (CDI). Editing modifies IQ to MR and inhibits calmodulin binding. Intracellular calcium increases in the absence of CDI.

Calmodulin (CaM) binds to the IQ domain located at the C-terminus of the pore forming α1 subunit. The formation of a Ca^2+^-CaM complex results in CDI (Tadross et al., 2008). Calcium binding to the N- and C-terminal CaM lobes can induce distinct channel regulation (Dick et al., 2008). Recently, editing of the core sequence of the IQ domain of Ca_v_1.3 by ADAR2 has been discovered. The core sequence comprises of the 4 amino acids IQDY. Upon editing different isoforms such as MQDY, IRDY, MQDC, MRDY, MRDC, or IQDC can be generated (see Figures 1 and 4). This editing event is restricted to the central nervous system (CNS; Huang et al., 2012). Additionally, the pattern of editing is developmentally regulated. It is negligible at e14 and prominently increases at p4. RNA editing of the IQ domain shows spatial distribution being highest at the frontal cortex and hippocampus (Huang et al., 2012). The MQ and IR versions show weaker CDI while the MR variant exhibits up to 50% reduction of CDI and faster recovery from inactivation. Reduction in CDI consequently increases the cellular calcium load (see Table 1).

## Serotonin 2C Receptor

The mammalian 5-hydroxytryptamine receptor subtype 2C (5-HT_2c_) is found widely distributed in the CNS. 5-HT_2c_R belongs to the G-protein-coupled receptor superfamily that stimulates phospholipase C (PLC) activity (Hoyer et al., 2002). The 5-HT_2c_ receptor interacts with the multi PDZ-domain bearing protein (MPDZ). Both these proteins co-localize on the apical membrane of choroid plexus cells (Drago and Serretti, 2009). The pre-mRNA encoding serotonin receptor 5-HT_2c_ is edited at five sites termed A, B, C, D, and E. Editing can lead to the exchange of three amino acids that are located within the second intracellular loop of the receptor (see Figure 2). This region has been shown to be important for efficient G-protein coupling (Niswender et al., 1998). Editing at the five positions in RNA can, in principle, yield a combination of 32 different mRNAs which encode 24 different protein isoforms (Burns et al., 1997; Niswender et al., 1999). In mouse brain, however, only eight major receptor variants can be detected at significant levels. Also sequencing analysis of human brain samples only detected 12 possible isoforms derived from RNA editing. This suggests that not all possible combinations of editing do exist *in vivo* (Wang et al., 2000; Wahlstedt et al., 2009). Moreover, the repertoire of possible editing combinations varies throughout development (Wahlstedt et al., 2009). Editing at sites A and B is accomplished by ADAR1, sites C, D, and E, however, are preferentially deaminated by ADAR2 (Hartner et al., 2004).

Upon editing, reduced G-protein coupling is observed in the edited states (Burns et al., 1997; see Figure 1). Editing not only reduces the constitutive activity of the receptor but also diminishes agonist potency and calcium release (Price et al., 2001). At the cellular level, editing changes the surface expression of the receptor. The subcellular distribution of the receptor depends on β arrestin-2 interaction with inverse correlation to the constitutive activity of the receptor. Editing decreases the constitutive activity and enhances surface expression. The fully edited VGV isoform displays complete surface expression. The unedited INI isoforms exhibit endosomal accumulation whereas the edited VSV isoforms with moderate activity display vesicular and cell surface expression (Marion et al., 2004). Editing also modulates the expression of the receptor through splicing. Unedited 5-HT_2c_ transcripts result in a splice version that yields a shorter protein, while edited transcripts form the full length receptor (Flomen et al., 2004). However, the underlying factors resulting in alternative splicing are still not entirely clear. It was shown, for instance, that the human and mouse C/D box snoRNAs HBII-52 and MBII-52, respectively, can inhibit site C editing via base-pairing (Vitali et al., 2005). This base-pairing event also seemingly masks a silencer element important for the regulation of splicing (Kishore and Stamm, 2006).

The serotonin 2c receptors can also activate extracellular signal-regulated kinase (ERK) independent of G-protein coupling. Expression of the unedited INI isoform increases ERK1/2 phosphorylation in transfected HEK293 cells while expression of the edited VGV form decreases phosphorylation. However, this activation is significantly reduced upon β-arrestin depletion (Labasque et al., 2010). Editing also decreases downstream ERK signaling. Consistently, a shift toward the edited isoforms leads to reduced ERK signaling in prefrontal cortex of ADAR2 transgenic mice (Singh et al., 2011). Overexpression of ADAR2 and hyperediting of the 5-HT-_2c_ receptor is also correlated with depressive behavior (Singh et al., 2009).

Mice carrying either a completely unedited “INI” version or a completely edited “VGV” version of the 5-HT-_2c_ receptor have been generated (Kawahara et al., 2008). While the INI mice grow normally but are rather immobile in a forced swim assay, resembling a depressive behavior (Mombereau et al., 2010). It has also been shown that a decrease in the INI form of the receptor can lead to a decrease in ERK1/2 phosphorylation in transgenic ADAR2 mice (Singh et al., 2011). Aberrant ERK1/2 phosphorylation in turn is linked to depression and suicidal behavior as ERK1/2 plays a critical role in synaptic plasticity (Dwivedi et al., 2009).

Mice expressing the fully edited VGV version of the receptor, in contrast, have reduced fat mass, growth retardation, and high energy expenditure most likely due to hyperactivation of the sympathetic nervous system (Kawahara et al., 2008). Mutant mice with fully edited VGV isoforms have also been shown to display symptoms resembling those of the Prader–Willi syndrome (PWS; Morabito et al., 2010).

Thus, data from transgenic mice clearly demonstrate that the editing status of 5-HT_2c-_R can directly influence behavior underscoring the importance of RNA editing for the etiology of psychiatric disorders.

## Actin Organization by Filamins

Two actin cross-linking proteins Filamin A (FLNa) and Filamin B (FLNb) are amongst a group of newly identified mammalian editing targets (Levanon et al., 2005; Nishimoto et al., 2008; Li et al., 2009). The two 280 kDa proteins form homo- and heterodimers and mediate orthogonal branching of actin filaments (Fucini et al., 1997; Sheen et al., 2002; Popowicz et al., 2006). Mammalian filamins are built of 24 immunoglobulin (Ig) like repeats divided into two rod segments. Rod 1 consisting of repeats 1–15 interacts with actin filaments whereas rod 2 is built from repeats 16–23 and interacts with several proteins (Chen et al., 2011). Repeat 24 is required for dimerization. Actin reorganization is essential for cell motility and migration and is an important determinant in dendritic spine and synapse formation (Dillon and Goda, 2005; Popowicz et al., 2006). Depletion of FLNa leads to embryonic lethality with severe cardiovascular and bone development defects (Feng et al., 2006; Hart et al., 2006). Also FLNb deficient mice show defective microvasculature and bone malformation (Zhou et al., 2007). Editing of FLNa or FLNb leads to a conserved glutamine (Q) to arginine (R) codon exchange in repeat 22 (Li et al., 2009) that is developmentally regulated (Wahlstedt et al., 2009). Repeat 22 has been shown to be involved in the interaction with a broad range of proteins (Popowicz et al., 2006; see Table 1).

FLNa interacts with the C-terminus of the metabotrobic glutamate receptor mGlu5a, 5b, 7b, and 8a (see Figure 1). Moreover, low affinity binding was also detected for mGlu4a and mGlu7a. Repeats 21 and 22of FLNa harboring the edited amino acid represent the minimal region critical for this interaction (Enz, 2002). Editing may thus regulate this interaction, the potential consequences of which remain to be determined.

FLNa also interacts with potassium channel Kv4.2 at filipodial roots and shows overlapping expression in cortical and hippocampal neurons. A “PTPP” amino acid motif in Kv4.2 (AA 601–604) is critical for this interaction. Again, FLNa repeats 21–24 are involved in this interaction. Coexpression of filamin in heterologous cells enhances the whole cell current density by ∼2.7-fold most likely by properly positioning functional K_V_4.2 receptors at the cell surface (Petrecca et al., 2000).

Presenilins (PS) belong to a conserved protein family that were the first proteins identified responsible for familial Alzheimer disease (FAD; Nelson et al., 2010). Presenilins harbor eight transmembrane domains. PS1 and PS2 were identified in a yeast two hybrid assay to interact with repeats 21–24 of FLNa. A region between TM6 and TM7 of the presenilins is responsible for this interaction. The same loop harbors 14 different mutations that are associated with FAD. FLNa and PS1 co-localize in astrocytes (Zhang et al., 1998). Moreover, overexpression of PS1 in cultured HEK293 cells redistributes FLN from the cell periphery to the cytoplasm. The FAD-linked mutation PS1M146L induces FLNa expression (Lu et al., 2010). The FLNa PS1interactionis well conserved and could be physically and genetically demonstrated in *Drosophila melanogaster* (Guo et al., 2000).

FLNa also co-localizes with the neuronal microtubule associated protein Tau. Tau is involved in polymerization and stability of microtubules. Tau protein is abnormally phosphorylated and forms neurofibrillary tangles in the hippocampus in Alzheimer patients. It is believed that Tau induced FLNa depletion leads to actin network destabilization and consequently to synaptic loss (Feuillette et al., 2010).

The functional implication of editing-induced Q2341R amino acid exchange in repeat 22of FLNa is still unknown. However, it may have an effect on a broad range of interactions (Chen et al., 2011). One example is the interaction of FLNa with β-integrin. Repeat 21 cannot interact with β-integrin unless repeat 20 disassociates from it (Lad et al., 2007). Similarly, FLNa editing may change neuronal receptor organization as well as synaptic transmission by altering the interaction profile with binding partners.

## Cytoplasmic FMRP Interacting Protein 2 (CyFIP2)

CyFIP2 was identified as an interaction partner of the fragile X mental retardation protein (FMRP) in a yeast two hybrid screen. The region of interaction between CyFIP2 and FMRP overlaps with the FMRP dimerization site (Schenck et al., 2001). CyFIP2 also interacts with FMRP-related proteins FXR1P and FXR2P (Schenck et al., 2001). The CyFIP2 encoding pre-mRNA is primarily edited by ADAR2 introducing a single K320Eamino acid exchange in mouse and human CyFIP2 (Levanon et al., 2005; Nishimoto et al., 2008).

CyFIP2 is a member of the WAVE/SCAR complex and is involved in actin remodeling. It plays a pivotal role in neuronal wiring as it directly interacts with FMRP and Rac-1 (Schenck et al., 2003). Flies have a single *Cyfip* gene which is 67% identical to human CyFIP1 and CyFIP2. Mutant *Cyfip* flies display shorter synapses and profound axonal path finding, growth, and branching defects (Schenck et al., 2003, 2004). CyFIP2 is mainly involved in maintaining synaptic plasticity as it is involved in translational regulation impeded in fragile X mental retardation. In vertebrates like Zebrafish that harbor both CyFIP1 and CyFIP2, *cyfip2* mutants exhibit dorso-nasal axonal pathfinding defects (Pittman et al., 2010). RNAi of CyFIP2 in murine melanoma cells leads to aberrant lamellipodia proving the functionality of Cyfip2 in actin remodeling and cell motility (Steffen et al., 2004).

Editing at the K/E position of CyFIP2 increases during mouse brain development ranging from 4% at e15 to 75% editing at p21 (Wahlstedt et al., 2009). However, there seems a significant decline in CyFIP2 editing with age in human brain (Nicholas et al., 2010).

The biological significance of Cyfip2 editing is currently not clear. One possibility would be that the migratory behavior of cells is regulated by CyFIP2 by antagonizing Rac-1. However, interactions with FMR1 or the nucleo-cytoplasmic shuttling of Cyfip2 might equally be affected by editing. With the new discovery of modulation of ADAR by FMR1 in flies the possibility of a feed back loop of CyFip2 and ADAR regulation also appears possible as Cyfip antagonizes FMR1 in flies (Schenck et al., 2003).

## Hu Proteins

Hu proteins are RNA-binding proteins which play an essential role in neuronal differentiation and plasticity. HuB, HuC, and Hu D are neuron specific whereas HuR is associated with cell stress responses. Each Hu protein has three RNA recognition motifs (RRM1–3). Hu proteins preferentially bind to AU rich RNA elements (ARE) where they can act as RNA stabilizers and regulators of polyadenylation and translation (Mobarak et al., 2000; Zhu et al., 2006; Hinman and Lou, 2008). Recently, five editing sites were discovered in HuD and HuB in a bioinformatic screen of deep-sequencing data (Enstero et al., 2010). The functional implication of editing is unknown. However, it is likely that editing of Hu proteins can alter the landscape of the brain transcriptome (Paz-Yaacov et al., 2010).

## Nova-1

Recently, another RNA-binding protein, NOVA-1, was found to be edited (Irimia et al., 2012). NOVA-1 is a key regulator of alternative splicing of RNAs encoding synaptic proteins involved in neuronal activity in the CNS. NOVA-1 binds pre-mRNAs in a sequence dependent manner and diversifies proteins by splicing regulation. *Nova-1* null mice die postnatally from motor neuron death due to spinal and brainstem neuron apoptosis (Jensen et al., 2000). The splicing regulation by NOVA-1/2 is well conserved from mammals to insects. Both Nova-1/2 and the *Drosophila melanogaster* ortholog PASILLA (PS) binds to YCAY enriched regions located upstream of repressed exons and downstream of activated exons (Brooks et al., 2011). RNA editing increases the Nova-1 half life by decreasing its susceptibility to proteasome degradation (Irimia et al., 2012). This stabilization of Nova-1 by RNA editing can create another layer of complexity in diversification of brain specific transcripts.

Dysregulation of A to I editing has been found associated with a number of diseases, ranging from mental disorders to cancers (Paz et al., 2007). The following sections will give an overview on diseases that are strongly influenced by ADAR-mediated editing.

## Astrocytoma

This glial cell tumor is classified on the basis of malignancy into four grades (I–IV). Glioblastomamultiforme (GBM) is a grade IV tumor with a survival rate of less than 18 months in children and adults (Stupp et al., 2005). Glial cells respond to external stimuli via neuronal receptors (Bergles et al., 2000; Gallo and Ghiani, 2000). Hypoediting of GluA2 at the Q/R site has been observed in GBM leading to increased Ca^2+^ influx and activation of the Akt pathway through phosphorylation (Ishiuchi et al., 2007). Also in pediatric astrocytoma the malignancy increases with a decrease in editing. GBM cells show strong migratory activity which is reduced upon ADAR2 expression. Furthermore a decrease in GluK2 editing at the I/V and Y/C sites is observed in different brain regions (Cenci et al., 2008). Since both GluA2 and GluK2 are edited by ADAR2, ADAR2 overexpression strongly inhibits cell proliferation and slows down the cell cycle. Mutation in the ADAR2 deaminase domain does not affect tumor malignancy proving the necessity of editing in tumor progression (Cenci et al., 2008). In this type of tumor ADAR2 is expressed at a normal level, while ADAR1 and ADAR3 are overexpressed leading to the assumption that higher concentrations of ADAR1 and ADAR3 may inhibit the activity of ADAR2 (Cenci et al., 2008).

In pediatric astrocytoma high levels of interferon induced ADAR1 p150 are found. Overexpression of ADAR1 might again interfere with ADAR2 activity (Chen et al., 2000; Cenci et al., 2008).

## Amyotrophic Lateral Sclerosis

Amyotrophic Lateral Sclerosis (ALS) is characterized by slow degeneration of upper and lower motor neurons with a consequent loss of voluntary movement (Rothstein, 2009). Different mechanisms are proposed to be the underlying causes of this disease. Decreased editing at the Q/R site leading to increased Ca^2+^ influx has been observed in mice displaying late onset ALS (Kuner et al., 2005). The editing efficiency at the GluA2 Q/R site also decreases dramatically in ALS patients (Kawahara et al., 2004). Consistent with reduced Q/R site editing, a significant decrease in ADAR2 expression has been observed in spinal motor neurons of ALS patients (Hideyama et al., 2011). However, no decline in editing of Q/R in upper motor neurons was observed.

Additionally, the flip-flop alternative splicing pattern of GluA2, downstream of the R/G editing site is pushed toward flip-bearing transcripts in ALS patients (Kawahara and Kwak, 2005). The flip form of GluA2 promotes assembly of slowly desensitizing AMPA receptors (Tomiyama et al., 2002).

Clearing of glutamate from the synaptic cleft is accomplished through glutamate transporters that prevent repeated firing and excitotoxicity. The astroglial EAAT2 glutamate transport is responsible for clearing glutamate from the cleft. ALS patients show 50% decreased EAAT2 protein levels as editing generates a cryptic polyadenylation site leading to intron 7 retention (Flomen and Makoff, 2011). Depletion of EAAT2 leads to neuronal death in transgenic mice (Rothstein et al., 1996).

## Prader–Willi Syndrome

The Prader–Willi locus is genomically imprinted and only expressed from the paternally inherited chromosome, while the maternal copy is transcriptionally silenced (Constancia et al., 2004). Loss of expression or mutation of the paternal 15q11–q13 locus therefore leads to the formation of the Pader–Willi disease phenotype. Patients have growth defects in both sexes due to growth hormone deficiency, and cognition problems (Butler, 2011). Amongst several other transcripts the small C/D box snoRNA MBII-52 is located within the Prader–Willi locus. This snoRNA contains 18 nucleotides that are complementary to the editing site C of the serotonin 5-HT_2c_ receptor. When expressed in nucleoli the 5-HT_2c_ pre-mRNA can even be targeted for 2′-O-methylation (Vitali et al., 2005). Loss of MBII-52 causes an increase in editing. Mice with a deleted PWS imprinted control region show enhanced locomotor activity and aberrant discriminative behavior (Doe et al., 2009). Altered 5-HT_2C_R editing can also lead to phenotypes that mimic PWS. Mice expressing the fully edited VGV form of the serotonin receptor also exhibit PWS-like phenotypes such as hyperphagia, hypotonia, increased metabolism, and slim stature (Morabito et al., 2010). Molecularly, this isoform exhibits blunted G-protein coupling, reduced constitutive activity and enhanced serotonergic neurotransmission possibly as a consequence of increased surface expression (Kawahara et al., 2008; Morabito et al., 2010).

## Transient Forebrain Ischemia

Cerebral ischemia in CA1 pyramidal neurons is caused by reduced oxygen supply, primarily as a consequence of heart attacks or occlusions of arteries. Neuronal damage is caused due to increased Ca^2+^ influx because of increased GluA2^Q^ expression (Liu et al., 2004). Increase in calcium activates Cdk5 which phosphorylates NMDA receptors (Liu et al., 2004). Phosphorylation, in turn, prolongs opening of NMDA receptors which can activate nitric oxide synthase leading to the formation of toxic peroxynitrite that induces neuronal death (Fiskum et al., 1999; Bossy-Wetzel et al., 2004). During experimental induction of ischemia in rat brain ADAR2 expression is reduced. Consistently, recovery from ischemia can be accomplished through increased ADAR2 expression (Peng et al., 2006).

Downregulation of R/G site editing has been observed during spinal cord injury (SCI). Reduced editing at this site may limit cell death progression by suppressing postsynaptic excitation. Thus, editing might influence recovery after SCI (Barbon et al., 2010). Reduced editing at the R/G site was also observed in rat prefrontal cortex upon treatment with phencyclidene (PCP) that instigates schizophrenia like behavior (Barbon et al., 2007).

## Epilepsy

Epilepsy is a common neurological disorder characterized by seizures caused by neuronal hyperexcitability (Bozzi et al., 2012). Decreased editing of the AMPA receptor Q/R site leads to calcium permeable channels. Mice heterozygous for an editing deficient GluA2 allele develop seizures and die at 3 weeks of age while complete absence of GluA2 expression does not provoke seizures (Brusa et al., 1995).

Increased editing at the R/G site of the GluA2 transcripts and also of K_v_1.1 have also been linked to seizures (Vollmar et al., 2004). Editing at the R/G site enhances glutamate response of the receptor and modulates neuronal excitability (Lomeli et al., 1994). The editing-induced I/V change in K_v_1.1 channels lies in the S6 segment. This is the target site of many drugs blocking the channel (Decher et al., 2010). Interestingly, the Kv channel blocker 4-aminopyridine (4-AP) also induces seizure like events in rats. RNA editing, in turn, reduces the affinity of 4-AP and serves as a compensatory mechanism against epileptic seizures (Streit et al., 2011).

## Psychiatric Disorders

Changes in the editing pattern of 5-HT_2C_ pre-mRNA have been linked to different psychiatric disorders such as schizophrenia, depression, and bipolar disorder (Table 1). Editing leads to reduced G-protein activation resulting in decreased basal activity (Niswender et al., 1999). However, the observed correlations do not allow a clear-cut conclusion. Sample sizes are typically small and the investigated samples are rarely well controlled and matched, therefore giving a heterogeneous picture. For instance, overexpression of the edited VSV receptor isoform has been observed in patients suffering from schizophrenia and bipolar disorders (Dracheva et al., 2008). Previously, in two different studies on suicide victims suffering schizophrenia, a significantly under edited B site and a hyper edited A site has been observed (Niswender et al., 2001; Sodhi et al., 2001). Analysis on suicide victims suffering major depression, in contrast, revealed an increase in editing at the C and C′ site accompanied by decreased D site editing. Treatment with fluoxetine, a serotonin selective uptake inhibitor, causes opposing effects on editing of these sites indicating site specific serotonin dependent regulation (Gurevich et al., 2002). Deregulation of A to I editing in schizophrenia and bipolar disorder (type I) patients and underediting of I/V site in GRIK2 resulting in high calcium influx has also been related to over expression of ADAR2 isoforms with diminished catalytic activity (Silberberg and Ohman, 2011; Silberberg et al., 2012). However, increase in ADAR1 expression has also been suggested as an inhibitor of ADAR2 activity (Simmons et al., 2010).

## Outlook

Current studies on RNA editing have clearly shown that adenosine deamination is most abundant in the CNS where it plays a major role in the diversification of the transcriptome. Three major processes seem to be primarily affected by A to I editing: first, many receptors and channels are modulated in their primary response and sensitivity to stimuli. Second, in many cases receptor assembly and retention in the ER seems to be affected by RNA editing. Finally, cytoskeletal components required for both outgrowth of neurons but also to the structuring of the cortical cytoskeleton and the anchoring of receptors is affected by RNA editing. It is one of the challenges to understand how these three processes are interconnected possibly being regulated through neuronal activity that may feed back on the process of RNA editing itself.

## Conflict of Interest Statement

The authors declare that the research was conducted in the absence of any commercial or financial relationships that could be construed as a potential conflict of interest.
